# Sensations of Starving

**Published:** 1874-06

**Authors:** 


### SENSATIONS OF STARVING.

FOR the first two days through which a strong and healthy man is doomed to exist upon nothing his sufferings are perhaps more acute than in the remaining stages—he feels an inordinate, unspeakable craving at the stomach night and day. The mind runs upon beef, bread and other substantials, but still, in a great measure, the body retains its strength. On the third and fourth days, but especially on the fourth, this incessant craving gives place to a sinking and weakness of the stomach, accompanied by a nausea. The unfortunate sufferer still desires food, but with a loss of strength he loses that eager craving which is felt in the earlier stages. Should he chance to obtain a morsel or two of food, he swallows it with a wolfish avidity; but five minutes afterwards his sufferings are more intense than ever. He feels as if he had swallowed a living lobster, which is clawing and feeding upon the very foundation of his existence. On the fifth day his cheeks suddenly appear hollow and sunken, his body attenuated, his color is ashy pale, and his eyes wild, glassy and cannibalish. The different parts of the system now war with each other. The stomach calls upon the legs to go with it in quest of food; the legs, from weakness, refuse. The sixth day brings with it increased suffering, although the pangs of hunger are lost in an overpowering languor and sickness. The head becomes giddy—the ghosts of well-remembered dinners pass in hideous processions through the mind. The seventh day comes, bringing increasing lassitude and further prostration of strength. The arms hang lifelessly, the legs drag heavily. The desire for food is still left, to a degree, but it must be brought, not sought. The miserable remnant of life which still hangs to the sufferer is a burden almost too grievous to be borne; yet his inherent love of existence induces a desire still to preserve it, if it can be saved without a tax upon bodily exertion. The mind wanders. At one moment he thinks his weary limbs cannot sustain him a mile, the next, he is endowed with unnatural strength, and if there be a certainty of relief before him, dashes bravely and strongly forward, wondering whence proceeds his new and sudden impulse.

